# The physiological anti-hypertensive peptide catestatin and its common human variant Gly364Ser: differential cardiovascular effects in a rat model of hypertension

**DOI:** 10.1042/BSR20241433

**Published:** 2024-12-12

**Authors:** Jitesh Singh Rathee, Dhanya R. Iyer, Malapaka Kiranmayi, Samarasimha Reddy, V.V. Sureshbabu, Nitish R. Mahapatra

**Affiliations:** 1 Bio-Organic Division, Bhabha Atomic Research Centre, Mumbai 400085, India; 2 Department of Biotechnology, Bhupat and Jyoti Mehta School of Biosciences, Indian Institute of Technology Madras, Chennai 600036, India; 3 Department of Studies in Chemistry, Central College Campus, Bangalore University, Dr. B. R. Ambedkar Veedhi, Bangalore 560001, India

**Keywords:** blood pressure, cardioprotection, catestatin, human genetic variant, inflammation

## Abstract

Catestatin (CST), a 21-amino acids physiological peptide, has emerged as a key modulator of cardiovascular functions due to its anti-hypertensive and cardioprotective properties. However, the ramifications of the most common human variant of CST (*viz*., Gly364Ser) on cardiovascular pathophysiology remain partially understood. In this study, hypertension was induced in uninephrectomized rats by treatment with deoxycorticosterone-acetate and sodium chloride (DOCA-salt). The DOCA-salt-induced hypertensive (DSHR) animals were then intraperitoneally administered with either CST wild-type (CST-WT) or 364Ser variant (CST-Ser) peptide. CST-Ser was profoundly less effective than CST-WT in rescuing the elevated systolic blood pressure [from ∼211 mmHg to ∼176 mmHg, *p* < 0.0001 (CST-Ser) *versus* ∼116 mmHg, *p* < 0.0001 (CST-WT)] and heart rate [from ∼356 bpm to ∼314 bpm, *p =* 0.66 (CST-Ser) *versus* ∼276 bpm, *p =* 0.02 (CST-WT)]. CST-Ser also showed diminished effects in lowering diastolic blood pressure and mean arterial pressure in the DSHR animals. Furthermore, CST-Ser was inefficient/markedly less potent in rescuing the impaired contractile and diastolic function in DSHR animals [improvements in the contractility index by ∼22 s^–1^ (CST-Ser), *p* = 0.15 *versus* by ∼84 s^–1^ (CST-WT), *p* < 0.0001 and decrease in end-diastolic pressure by ∼4 mmHg (CST-Ser), *p* = 0.015 *versus* by ∼14 mmHg (CST-WT), *p* < 0.0001]. Moreover, CST-Ser exerted less potent anti-inflammatory effects on the DSHR hearts than CST-WT. These findings are in concordance with the elevated systolic/diastolic blood pressure observed in Ser variant carriers from various human populations. This study provides compelling evidence for the diminished anti-hypertensive and cardioprotective effects of the CST-Gly364Ser variant.

## Introduction

Hypertension remains one of the leading risk factors for cardiovascular diseases (CVDs) and global mortality [1] (https://www.healthdata.org/research-analysis/library/risk-factors-driving-global-burden-disease). According to the World Health Organization, globally, approximately 1.28 billion people have hypertension (https://www.who.int/news-room/fact-sheets/detail/hypertension). The Mosaic Theory posited that hypertension is a multifactorial condition characterized by a complex interplay between genetic and environmental factors [2].

Chromogranin A (CHGA), a secretory protein localized primarily in the secretory granules of the neuronal and neuroendocrine systems [3], is a key molecular player in the pathogenesis of hypertension. CHGA undergoes post-translational modifications to give rise to several bioactive peptides [4], the most well-studied of which is catestatin (CST). Targeted ablation of the *Chga* gene in mice resulted in elevated blood pressure (BP), which was mitigated upon administration of CST [5]. Plasma CST levels have been found to be diminished not only in hypertensive subjects [6], but also their normotensive offspring [7], suggesting that low CST levels augment hypertension risk. A recent study demonstrated the modulatory effects of CST on mitochondrial energetics in heart failure with preserved ejection fraction (HFpEF). Interestingly, serum CST levels were higher in patients with HFpEF and correlated with brain natriuretic peptide and left ventricular (LV) end-diastolic filling pressure [8]. Naturally-occurring variants in the CST region (*viz*., Tyr363Tyr, Gly364Ser, Gly367Val, Pro370Leu, and Arg374Gln) have been identified in several world populations [9,10]. Among these human variants of CST, the Gly364Ser variant is reasonably conserved across different species, but its frequency varies significantly in different ethnic populations [11]. The Gly364Ser variant was found to be associated with an enhanced risk for hypertension in an Indian population [11]. Moreover, the Ser allele was also associated with higher systolic blood pressure (SBP) and pulse pressure (PP) in a Japanese population [12]. The CST-Ser variant peptide was less potent in stimulating nitric oxide (NO) production in human umbilical vein endothelial cells, and exhibited altered interactions with nicotinic acetyl cholinergic receptor (nAChR) and β-adrenergic receptor, thus altering the risk for hypertension [10,11].

Several rodent models are employed to delineate the molecular mechanisms underlying the complex pathophysiology of hypertension [13]. Administration of deoxycorticosterone-acetate (DOCA) along with sodium chloride (NaCl) in rats elicits a marked elevation in their SBP and diastolic blood pressure (DBP) [14], in addition to impairing their renal function [15]. DOCA-salt-treated animals display enhanced sympathetic activity and altered neurohumoral responses, which are typically observed in hypertension. These animals also exhibit cardiovascular remodeling and alterations in the peripheral vasculature, such as cardiac and vascular hypertrophy, fibrosis, conduction abnormalities, and endothelial dysfunction [16,17].

There are scattered reports demonstrating the role of CST in hypertension in rodent models. CST-KO mice were hypertensive, which was rescued upon CST supplementation [18]. CST treatment seemed to have a protective effect on target organs in spontaneously hypertensive rats (SHR) [19]. Blunted Frank-Starling responses in SHR as compared to their normotensive counterparts (Wistar Kyoto Rats, WKY) were improved by CST administration [20]. However, there is no systematic study which compares the potencies of the CST-WT and CST-Ser peptides in modulating cardiac performance in rodent models of hypertension.

In this study, we sought to assess the effect of the CST-Ser variant on cardiovascular function in uninephrectomized DOCA-salt-treated hypertensive rats (DSHR). The variant peptide (CST-Ser) displayed a markedly diminished effect than the wild-type peptide (CST-WT) in rescuing BP and providing cardioprotection to DSHR animals. These findings provide mechanistic insights into the inter-individual variations in BP and cardiac parameters, and may help in the development of novel cardiovascular prognostic strategies.

## Materials and methods

### Synthesis and purification of peptides

The wild-type CST (CST-WT: SSMKLSFRARAYGFRGPGPQL) and variant CST (CST-Ser: SSMKLSFRARAY**S**FRGPGPQL; the variant serine residue is shown in bold) peptides were synthesized using the Fmoc-solid-phase method. A schematic outlining the process is depicted in Figure S1. Briefly, both the peptides were synthesized using the Fmoc-Leu-Wang resin (0.5 mmol/g) with the scale of synthesis being 0.4 mmol. Coupling conditions used were: 4 equivalents of amino acid, 0.38 equivalent of HBTU, and 8 equivalents of DIPEA. All the amino acid couplings, except arginine, were performed at room temperature for 60 min. Double couplings were needed to achieve the complete insertion of arginines into the sequence. Fmoc removal was carried out by using 20% piperidine in DMF for 10 min and 12 min. All the coupling cycles were monitored by Kaiser test. After complete chain assembly, the resin-bound peptide was released using a mixture of TFA: TIS: H_2_O (95:2.5:2.5) for about 2.5-3 h. The resin was filtered off and the cleavage cocktail was reduced. The peptide was then crashed out from the cleavage cocktail with excess of cold ether. The mixture was cooled to 0°C for 1.5 h before the crude peptide was filtered off, redissolved in H_2_O/MeCN and lyophilized. The peptide was then purified using reverse phase-high performance liquid chromatography (RP-HPLC) with a gradient of 5-50% ACN in 30 min with 15 mL flow.

For synthesis of the CST-Ser peptide, Ser was incorporated at position 13 instead of the Gly residue. The above-described protocols were used to synthesize the CST-Ser peptide. A schematic outline of the synthesis steps followed is depicted in Figure S1.

The purity (>95%) of the peptides was assessed from the RP-HPLC profiles, and their identities were confirmed by high-resolution mass spectroscopy (Figures S2 and S3). Calculated molecular mass of CST-WT (C_104_H_164_N_32_O_27_S): 2325.2164; observed molecular mass: 2326.800. Calculated molecular mass of CST-Ser13 (C_105_H_166_N_32_O_28_S): 2355.2270; observed molecular mass: 2356.469.

### Animals

The animal study protocol was approved by Bhabha Atomic Research Centre (BARC) Animal Ethics Committee (project no BAEC 19/18). The study was conducted in BARC, Mumbai, India in strict adherence to the ethical guidelines laid down by the European Convention for the Protection of Vertebrate Animals used for Experimental and Other Scientific Purposes, as well as, the Committee for the Purpose of Control and Supervision of Experiments on Animals, constituted by the Animal Welfare Division, Government of India.

Six to eight weeks-old male Wistar rats, weighing around 200-250 g, were obtained from the BARC animal house facility, Mumbai, India. The animals were subjected to unilateral nephrectomy. The rats were anesthetized by means of an intra-peritoneal injection of ketamine, diazepam, and xylazine (70 mg/kg, 2 mg/kg, and 5 mg/kg, respectively), following which a lateral abdominal incision was made and the left renal vessels and the ureter were ligated. The left kidney was then removed and weighed, and the incision was sutured. All the subsequent experiments were performed on these rats (UNX). The body weights, food and water intake of the animals were monitored everyday throughout the duration of the study.

### Development of the DOCA-salt hypertensive model and treatment with CST peptides

The animals were divided into four groups (*n* = 8 each). The sham-treated animals served as the negative control (UNX group). The second group received a subcutaneous injection (0.4 mL) of 24 mg/kg DOCA on every fourth day, along with drinking water containing 1% NaCl for 28 days, to allow for development of hypertension (DSHR group); this group served as the positive control. DOCA was dissolved in dimethyl formamide. Negative control animals were injected with equal volume (that is, 0.4 mL) of dimethyl formamide without DOCA. The third and fourth groups were also given DOCA and salt treatments, but in addition were also treated with intraperitoneal injections of 1 µM (6 µg/kg body weight) of the wild-type (CST-WT group) and variant (CST-Ser group) CST peptides, respectively, every day from day 14 to day 28.

### Non-invasive, intra-arterial, and intra-ventricular BP measurements

The BP of animals in the control and treated groups was measured on 28^th^ day. SBP of the normal and UNX rats given only 1% saline water were also compared. Non-invasive measurements of SBP entailed the rats being subjected to light sedation with ketamine (50 mg/kg) and diazepam (2 mg/kg) intraperitoneally, followed by ten minutes of sleep, and then measurement of SBP using an MLT1010 Piezo-Electric Pulse Transducer (AD Instruments, Sydney, Australia) and an inflatable tail-cuff, connected to a NIBP controller and PowerLab data acquisition unit (PL3508/P (AD Instruments) using LabChart pro software 8.0 (AD Instruments). Ten readings were taken for each rat, and they were placed under a 60W light bulb to keep them warm during sedation. The rats were trained for tail-cuff measurements prior to actual recording of readings.

The intra-arterial measurements of BP were carried out on the 28^th^ day using a closed-chest approach (right/left carotid artery catheter insertion) following previously standardized protocol [21] with slight modifications. In brief, the neck of the anesthetized animal was shaved and opened carefully to expose the trachea. The visible trachea was then put aside and carotid artery was probed beneath the side of trachea. After the carotid artery was exposed, it was carefully separated from the vagus nerve ensuring that the nerve was not damaged. The artery was cannulated with Millar micro tip SPR 320 pressure sensitive transducer (Millar Instruments Inc., Houston, TX, USA) and securely tied. After the first trace was observed the catheter was pushed further into the aorta to see the notch trace. The catheter was pushed further through the aorta to ventricle, till the dicrotic notch in the trace disappeared and ventricular trace appeared. The reading was recorded for 5 min with PowerLab data acquisition system PL3508/P (AD Instruments). LabChart pro software 8.0 (AD Instruments) was used to analyze the data.

### Electrocardiogram (ECG) and heart rate variability (HRV) measurements

ECG measurements were taken on the 28^th^ day of the experiment, with needle electrodes using BioAmp and PowerLab data acquisition system PL3508/P (AD Instruments). The results are an average of 10-min readings each. HRV measurements were made using the frequency domain method while taking the ECG as the base reading. Lab Chart pro software 8.0 (AD Instruments) was used for data analysis.

### Histological analysis of cardiac tissues

Animals from all the four experimental groups were euthanized using sodium thiopentone (100 mg/kg, i.v.) and the organs were harvested. The cardiac tissues were sectioned after fixing in 10% formaline-buffered saline solution for 7 d, dehydrated, and embedded in paraffin blocks. Thin sections (10 mm) of LV were cut and stained with haematoxylin and eosin (H&E) to assess the inflammatory status in these animals The tissue sections were graded as: *NAD* (no abnormalities detected), *minimal* (1/+), *mild* (2/++), *moderate* (3/+++), and *marked* (4/++++), depending on the extent of lymphocyte/leucocyte infiltration along with sarcolysis in the necrotic area.

### Data representation and statistics

The experimental data, representative of at least three independent measurements from eight animals per group, have been expressed as mean ± standard error of mean. Statistical analysis was performed using one-way analysis of variance followed by multiple comparisons *post-hoc* tests, as applicable, and visualized using Prism version 8 (GraphPad Software, San Diego, CA, USA). Statistical significance has been represented as *p < 0.05, **p < 0.01, ***p < 0.001, and ****p < 0.0001.

Meta-analysis was performed using the Meta-Analyst tool (http://www.cebm.brown.edu/openmeta/index.html#) For the Indian and Japanese cohorts, the individuals were grouped as Gly/Gly and Gly/Ser+Ser/Ser. Data for the Indian, Japanese, and Californian populations were obtained from Kiranmayi *et al*. [11], Choi *et al*. [12], and Rao *et al*. [22], respectively. Additionally, data was also mined from the following genome-wise association studies (GWASes): GoDarts Affymetrix GWAS for SBP and GoDarts exome chip analysis for DBP [23], Blood pressure traits 2017 GWAS (African ancestry), and AMP T2D-GENES quantitative trait exome sequence analysis (Hispanic) [24]. Beta coefficients from the GWASes were obtained from the Common Metabolic Diseases Knowledge Portal (cmdkp.org) (rs9658667 variant page. 2024 Sept 5; https://hugeamp.org/variant.html?variant=rs9658667), and GWAS ATLAS (https://atlas.ctglab.nl/). The standard errors were calculated using the Meta-Analyst tool and the forest plot was visualized using Prism 8.

## Results

### Meta-analysis of the association of the human CST Gly364Ser variant with BP

**Figure 1:**
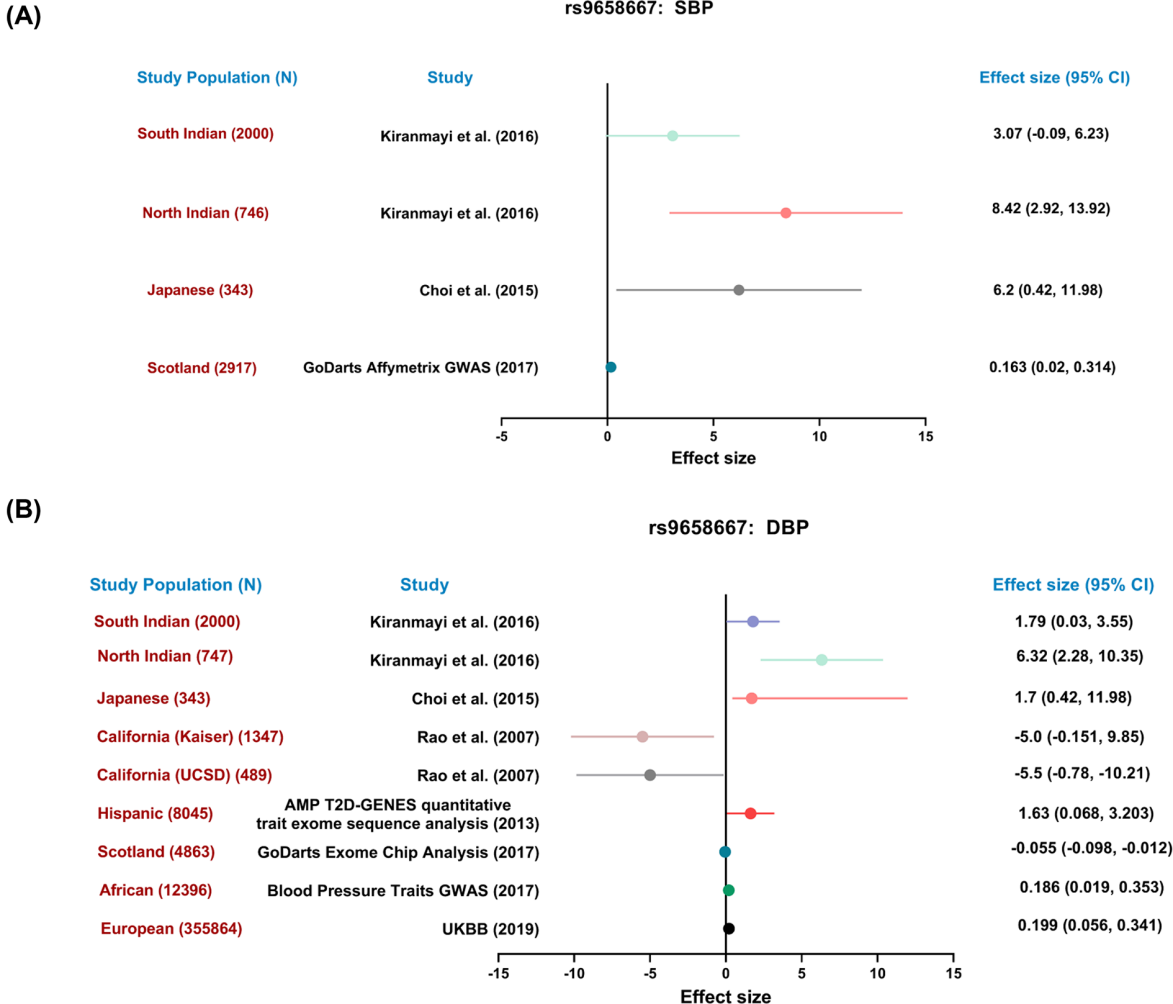
Meta-analysis of the effect of the 364Ser allele on blood pressure A forest plot depicting the overall effect of the 364Ser allele on (**A**) SBP and (**B**) DBP in several populations. For the Indian, Japanese, and Californian studies, the mean differences were considered as the metric of effect size. For the GWASes, beta coefficients were used as a measure of effect size. SBP: systolic blood pressure, DBP: diastolic blood pressure.

Previous studies by us and others showed association of the most common variant of CST (*viz*., Gly364Ser) with the risk of hypertension in some human populations [11,12]. Here, we carried out a meta-analysis to assess the effect of the CST Ser allele on BP, across several global populations. In concordance with these previous studies the Ser allele showed, *in general*, higher SBP and DBP levels (indicated by positive effect sizes) in several ethnic populations (Figure 1).

### Differential effects of CST-WT and CST-Ser peptides on BP in DSHR animals

**Figure 2:**
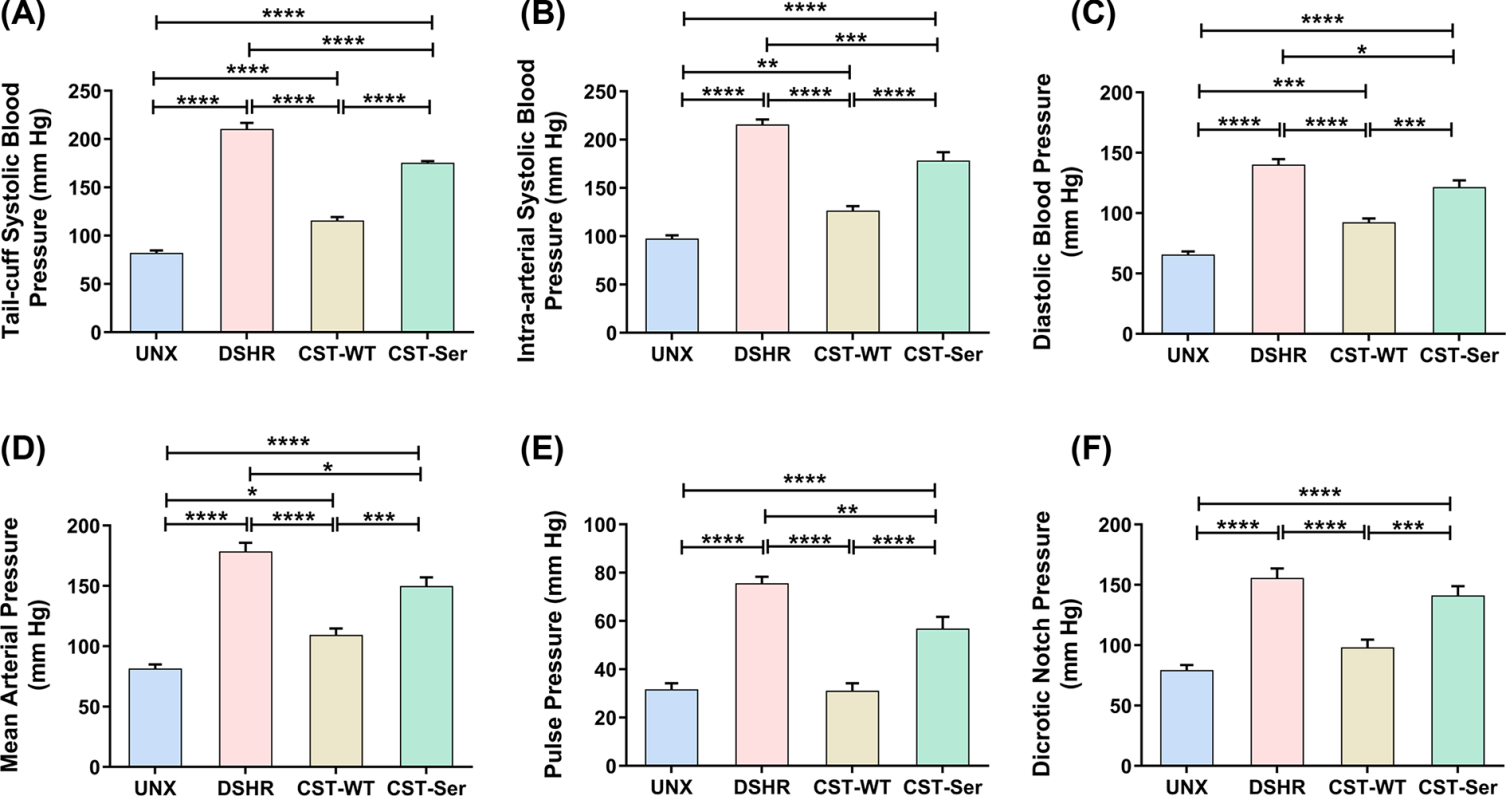
Non-invasive and intra-arterial measurements of blood pressure after administration of CST-WT and CST-Ser peptides Blood pressure was assessed in all animals on the 28^th^ day following intra-peritoneal administration of the CST peptides. The values are shown as mean ± SEM, and differences in these parameters among the experimental groups were analyzed using one-way ANOVA followed by Bonferroni's multiple comparisons *post-hoc* test. Systolic blood pressure was measured using the (**A**) non-invasive tail-cuff (*F* = 220.7, *p* < 0.0001) and (**B**) intra-arterial method (*F* = 84.68, *p* < 0.0001). Other intra-arterial measurements of blood pressure included (**C**) diastolic blood pressure (*F* = 61.96, *p* < 0.0001), (**D**) mean arterial pressure (*F* = 51.16, *p* < 0.0001), (**E**) pulse pressure (*F* = 40.11, *p* < 0.0001), and (**F**) dicrotic notch pressure (*F* = 28.98, *p* < 0.0001). UNX: control animals subjected to unilateral nephrectomy and administered with vehicle; DSHR: animals subjected to unilateral nephrectomy and DOCA-salt treatments; CST-WT: DSHR animals treated with CST-WT peptide; CST-Ser: DSHR animals treated with CST-Ser peptide. Number of animals per group = 8. The intra-arterial measurements are an average of 5-min readings. *p < 0.05, **p < 0.01, ***p < 0.001, and ****p < 0.0001.

BP measurements using the tail-cuff method revealed elevated SBP levels in the DSHR group than the UNX group [210.5 ± 6.2 mmHg (DSHR) *vs*. 82.2 ± 2.5 mmHg (UNX), *p* < 0.0001]. While intraperitoneal administration of CST-WT resulted in rescuing of BP to 115.7 ± 3.6 mmHg (*p* < 0.0001) CST-Ser-treated animals remained hypertensive (175.5 ± 1.7 mmHg, *p* < 0.0001) (Figure 2A).

The intra-arterial BP measurements were consistent with the tail-cuff BP measurements: the augmented SBP [215.8 ± 5.1 mmHg (DSHR) *vs.* 97.5 ± 3.5 mmHg (UNX), *p* < 0.0001] was markedly reduced upon administration of CST-WT (126.5 ± 4.6 mmHg, *p* < 0.0001), but administration of CST-Ser reduced the SBP to a much lesser extent (to 178.4 ± 8.5 mmHg, *p* = 0.0005) (Figure 2B). Similar trends were seen for DBP [92.4 ± 3.2 mmHg (CST-WT), *p* < 0.0001 and 121.5 ± 5.6 mmHg (CST-Ser), *p* = 0.02 *vs*. 140.2 ± 4.6 mmHg (DSHR)] (Figure 2C) and mean arterial pressure [MAP; 109.4 ± 5.4 mmHg (CST-WT), *p* < 0.0001 and 149.9 ± 7.2 mmHg (CST-Ser), *p* = 0.01 *vs*. 178.5 ± 7.2 mmHg (DSHR)] (Figure 2D). CST-WT treatment diminished the PP of the hypertensive rats to the level of the control group [31.7 ± 2.5 mmHg (UNX); 31.1 ± 3.1 mmHg (CST-WT), *p* < 0.0001 and 56.9 ± 4.8 mmHg (CST-Ser), *p* = 0.003 *vs*. 75.6 ± 2.7 mmHg (DSHR)] (Figure 2E). As compared to the DSHR condition, the dicrotic notch pressure declined significantly in rats treated with CST-WT [98.2 ± 6.4 mmHg (CST-WT) *vs*. 155.8 ± 7.8 mmHg (DSHR), *p* < 0.0001], but not in the case of CST-Ser treatment (141.3 ± 7.6 mmHg, *p* = 0.81) (Figure 2F).

### Effect of CST-WT and CST-Ser peptides on ECG measurements

**Figure 3:**
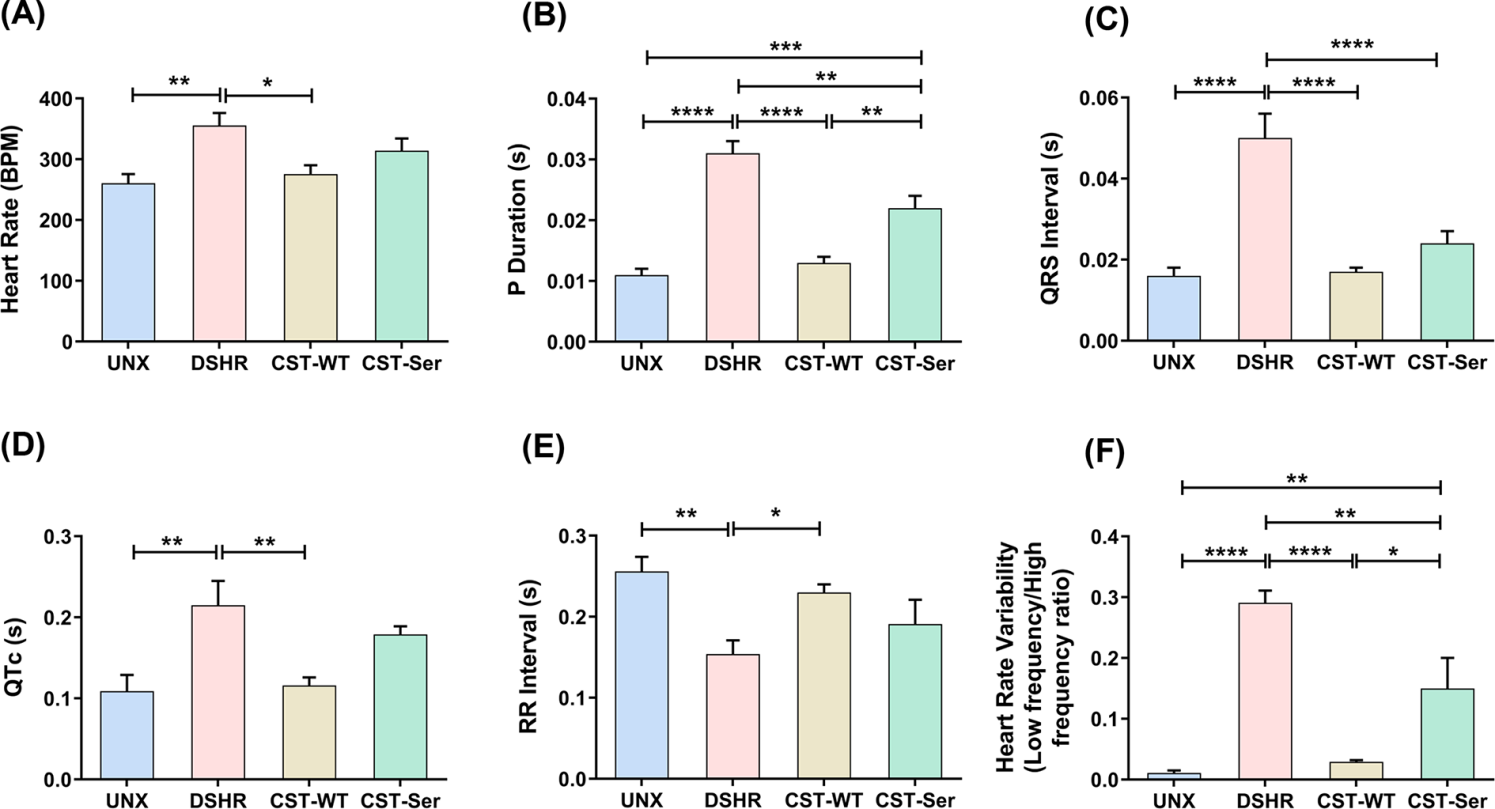
Effect of CST-WT and CST-Ser peptides on cardiac conduction and autonomic function Electrocardiogram readings were taken on the 28^th^ day following intra-peritoneal peptide administration. Ten-minute average readings are shown as mean ± SEM, and differences in these parameters among the experimental groups were analyzed using one-way ANOVA followed by Bonferroni's or Dunnett's multiple comparisons *post-hoc* test: (**A**) Heart rate (*F* = 5.748, *p* = 0.0034), (**B**) P-duration (*F* = 33.7, *p* < 0.0001), (**C**) QRS interval (*F* = 20.23, *p* < 0.0001), (**D**) QTc interval (*F* = 6.945, *p* = 0.0012), (**E**) RR interval (*F* = 4.954, *p* = 0.007), and (**F**) heart rate variability (*F* = 22.93, *p* < 0.0001). UNX: control animals subjected to unilateral nephrectomy and administered with vehicle; DSHR: animals subjected to unilateral nephrectomy and DOCA-salt treatments; CST-WT: DSHR animals treated with CST-WT peptide; CST-Ser: DSHR animals treated with CST-Ser peptide. Number of animals per group = 8. *p < 0.05, **p < 0.01, ***p < 0.001, and ****p < 0.0001.

High heart rates foreshadow the development of hypertension and associated conditions, such as metabolic syndrome [25]. The DSHR group displayed significantly higher heart rate than the UNX group (355.5 ± 20.5 bpm *vs*. 260.4 ± 15.2 bpm, *p* = 0.0045). While treatment with CST-WT lowered the heart rate (275.5 ± 14.8 bpm, *p* = 0.02) to the level of the UNX group, treatment with CST-Ser did not exhibit significant reduction (314.2 ± 19.8 bpm, *p* = 0.66) (Figure 3A).

Abnormal P-wave indices (P-wave duration and dispersion) are predictive of atrial fibrillation [26], as well as, the susceptibility of hypertensive patients to atrial fibrillation [27]. Elevation in the P-durations upon generation of the DOCA model [0.011 ± 0.001 s (UNX) *vs*. 0.031 ± 0.002 s (DSHR), *p* < 0.0001] declined after CST-WT and CST-Ser treatments [0.013 ± 0.001 s (CST-WT), *p* < 0.0001 and 0.022 ± 0.002 s (CST-Ser), *p* = 0.002 *vs*. DSHR] (Figure 3B), with a more prominent reduction in the CST-WT-treated animals.

Elevated BP levels prolong the QRS and QTc intervals [28,29]. Similar elevations in the QRS and QTc intervals were observed in the DSHR animals [0.05 ± 0.006 s (DSHR) *vs*. 0.016 ± 0.002 s (UNX), *p* < 0.0001; 0.215 ± 0.03 s (DSHR) *vs*. 0.109* ±* 0.02 s (UNX), *p* = 0.003, respectively], which subsequently diminished upon administration of CST-WT (QRS = 0.017 ± 0.001 s, *p* < 0.0001 and QTc = 0.116 ± 0.01 s, *p* = 0.007), but not CST-Ser (0.024 ± 0.003 s, *p* < 0.0001 and 0.179 ± 0.01 s, *p* > 0.99 *vs*. DSHR, respectively) (Figure 3C,D). CST-Ser did not have a significant impact on the RR interval [0.154 ± 0.02 s (DSHR) *vs*. 0.191 ± 0.03 s (CST-Ser), *p* = 0.43], while CST-WT elevated it significantly [0.23 ± 0.01 s (CST-WT), *p* = 0.032 *vs*. DSHR], to near control levels (0.256 ± 0.018 s; *p* > 0.99 vs. CST-WT) (Figure 3E). However, while CST-WT administration caused a profound reduction in the HRV, represented by the ratio of low frequency (LF)/high frequency (HF) [0.291 ± 0.02 (DHSR) *vs*. 0.029 ± 0.003 (CST-WT), *p* < 0.0001], CST-Ser was not as effective (0.15 ± 0.05, *p* = 0.006 *vs*. DSHR) (Figure 3F).

### Effect of CST-WT and CST-Ser peptides on myocardial contractility and LV diastolic function

**Figure 4:**
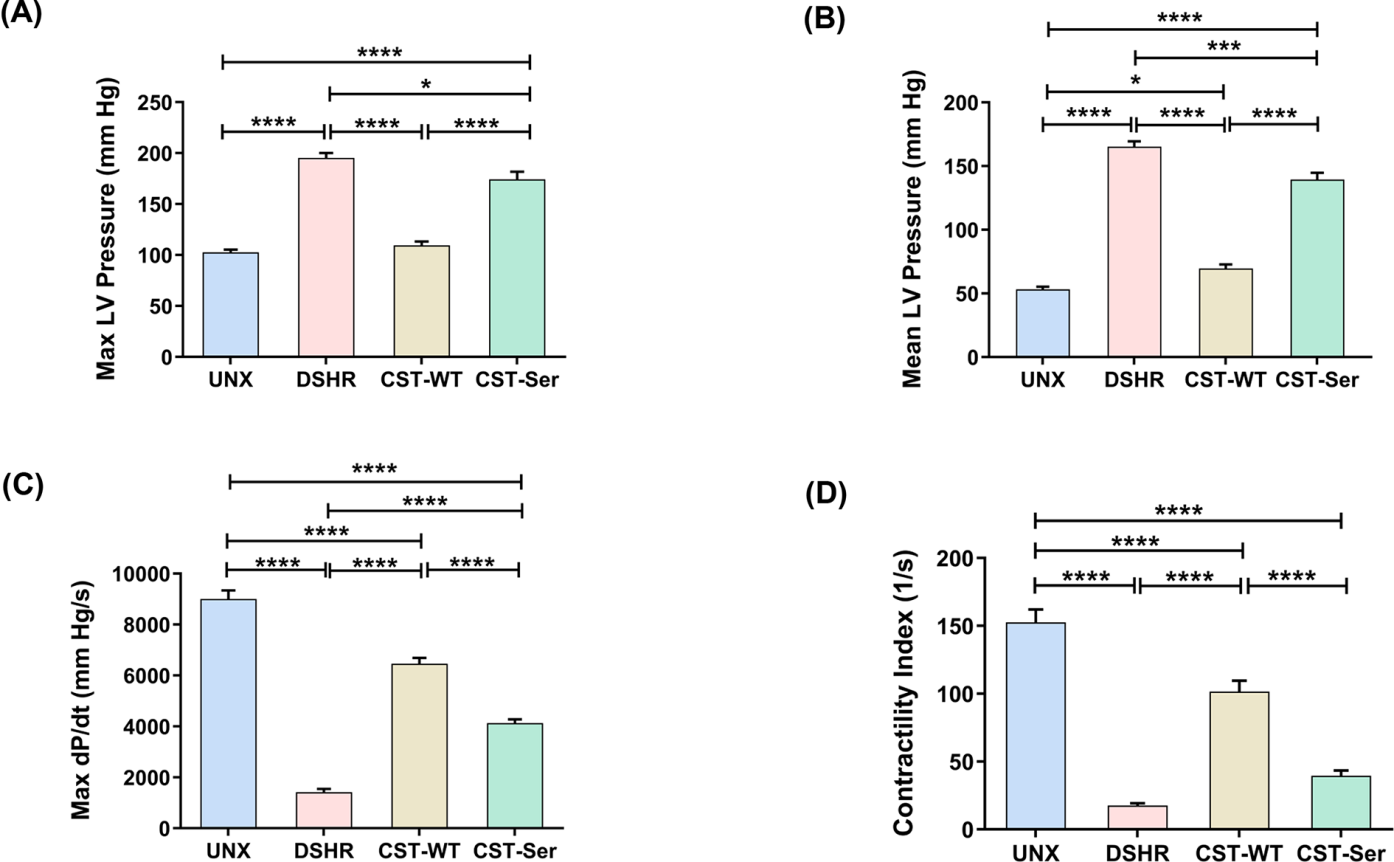
Impact of CST-WT and CST-Ser peptides on myocardial contractile function The contractile function of the experimental groups was assessed using the following parameters: (**A**) Max LV pressure (*F* = 86.1, *p* < 0.0001), (**B**) Mean LV pressure (*F* = 190.40, *p* < 0.0001), (**C**) Max dP/dT (*F* = 209.3, *p* < 0.0001), and (**D**) Contractility index (*F* = 86.32, *p* < 0.0001). All values have been represented as mean ± SEM and analyzed using one-way ANOVA followed by Bonferroni's multiple comparisons *post-hoc* test. UNX: control animals subjected to unilateral nephrectomy and administered with vehicle; DSHR: animals subjected to unilateral nephrectomy and DOCA-salt treatments; CST-WT: DSHR animals treated with CST-WT peptide; CST-Ser: DSHR animals treated with CST-Ser peptide. Number of animals per group = 8. *p < 0.05, **p < 0.01, ***p < 0.001, and ****p < 0.0001.

The contractile performance of the myocardium is assessed in terms of LV pressure and its derivative. (dP/dT)_max_ is the maximal rate of rise of LV pressure in the cardiac cycle, *i.e*., during ventricular systole, whereas (dP/dT)_min_ represents the peak negative value of rate of pressure change that occurs during the ventricular diastole [30,31]. The DSHR group exhibited an increase in the Max LV pressure [102.5 ± 2.8 mmHg (UNX) *vs*. 195.4 ± 4.8 mmHg (DSHR), *p <* 0.0001]. CST-WT caused a profound reduction in Max LV pressure (109.5 ± 3.8 mmHg *vs*. DSHR, *p* < 0.0001), while CST-Ser caused only a partial reduction (to 174.30 ± 7.4 mmHg *vs*. DSHR, *p* = 0.03) (Figure 4A). A similar trend was also observed in case of Mean LV pressure [165.3 ± 4.1 mmHg (DSHR) *vs*. 69.5 ± 3.3 mmHg (CST-WT), *p* < 0.0001 and 139.4 ± 5.4 mmHg (CST-Ser), *p* = 0.0004] (Figure 4B). The substantial reductions observed in the (dP/dT)_max_ values of the DSHR animals were significantly rescued upon administration of CST-WT [6458.4 ± 231.5 mmHg/s *vs*. 1421.2 ± 125.4 mmHg/s (DSHR), *p* < 0.0001], while CST-Ser was less efficacious (4132.5 ± 147.9 mmHg/s, *p* < 0.0001). LV contractility index (dp/dt_max_/P) (dσ*/dt_max_), a parameter used to evaluate myocardial contractility in the context of LV wall stress [32], was significantly blunted in the DSHR animals [17.53 ± 1.8 s^–1^ *vs*. 152.51 ± 9.5 s^–1^ (UNX), *p* < 0.0001]. There was a marked enhancement in the contractility index upon administration of CST-WT (101.5 ± 8.1 s^–1^ *vs*. DSHR, *p* < 0.0001), but not in the case of CST-Ser group (39.6 ± 3.8 s^–1^ *vs*. DSHR, *p* = 0.15).

**Figure 5:**
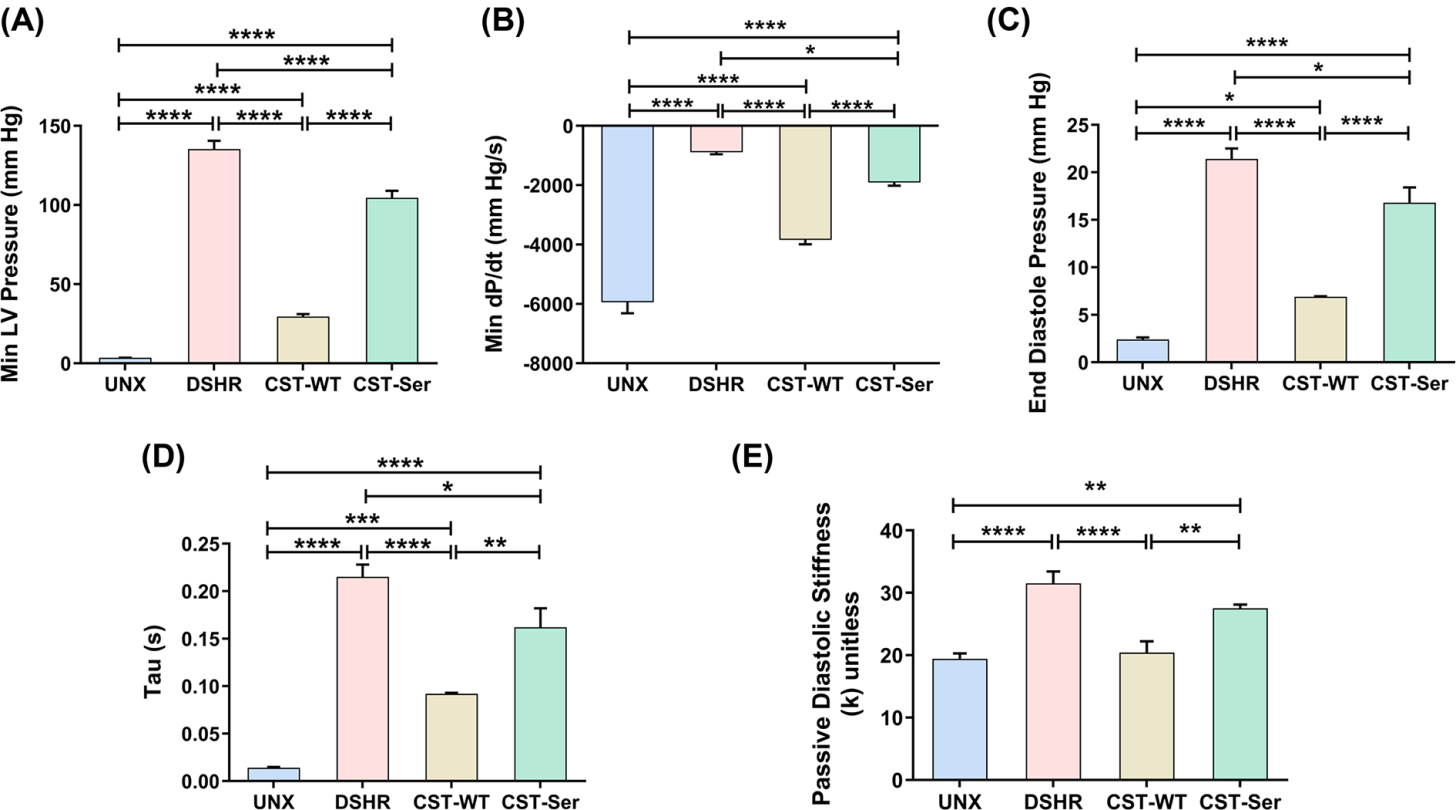
Effect of CST-WT and CST-Ser peptides on diastolic LV function Diastolic LV function was evaluated on the 28^th^ day following intra-peritoneal administration of the CST peptides, using the parameters: (**A**) min LV pressure (*F* = 315.3, *p* < 0.0001), (**B**) min dP/dT (*F* = 110.8, *p* < 0.0001), (**C**) end-diastolic pressure (*F* = 80.23, *p* < 0.0001), (**D**) relaxation time constant Tau (*F* = 53.26, *p* < 0.0001), and (**E**) passive diastolic stiffness (*F* = 16.73, *p* < 0.0001). The results have been represented as mean ± SEM and analyzed using one-way ANOVA followed by Bonferroni's multiple comparisons *post-hoc* test. UNX: control animals subjected to unilateral nephrectomy and administered with vehicle; DSHR: animals subjected to unilateral nephrectomy and DOCA-salt treatments; CST-WT: DSHR animals treated with CST-WT peptide; CST-Ser: DSHR animals treated with CST-Ser peptide. Number of animals per group = 8. *p < 0.05, **p < 0.01, ***p < 0.001, and ****p < 0.0001.

CST-WT was observed to be more potent than CST-Ser in the mitigation of LV diastolic dysfunction, as evidenced by a marked reduction of several deterministic parameters of LV function in the DSHR animals treated with CST-WT, as compared to those treated with CST-Ser. CST-WT was able to ameliorate the elevation in Min LV pressure [29.5 ± 1.5 mmHg *vs*. 135.3 ± 5.2 mmHg (DSHR), *p* < 0.0001] to a greater extent than CST-Ser (104.50 ± 4.4 mmHg, *p* < 0.0001) (Figure 5A). Similarly, CST-WT was more effective in the recovery of the (dP/dT)_min_ ratios than CST-Ser [–890.54 ± 65.2 mmHg/s (DSHR) *vs*. –3840.8 ± 152.4 mmHg/s (CST-WT), *p* < 0.0001 and –1906.7 ± 108.5 mmHg/s (CST-Ser), *p* = 0.01] (Figure 5B). Of note, although CST-WT treatment was not successful in improving the (dP/dT)_min_ ratio to the control level (–5940.77 ± 374.5 mm Hg/s), it exhibited a ∼4.3-fold increase, as opposed to the ∼2.1-fold increase by CST-Ser (with respect to DSHR), indicating enhanced efficacy of the CST-WT peptide with respect to the variant peptide in terms of cardiac performance.

The end-diastolic pressure was significantly higher in the DSHR animals [21.4 ± 1.1 mmHg *vs*. 2.4 ± 0.2 mmHg (UNX), *p* < 0.0001], but declined substantially upon treatment with CST-WT (6.90 ± 0.04 mmHg, *p* < 0.0001), but not CST-Ser (16.81 ± 1.6 mmHg, *p* = 0.015) (Figure 5C). A similar trend was also observed for Tau, the LV relaxation time constant [0.215 ± 0.013 s (DSHR) *vs*. 0.092 ± 0.001 s (CST-WT), *p* < 0.0001 and 0.162 ± 0.02 s (CST-Ser), *p* = 0.02] (Figure 5D). Passive diastolic stiffness, a measure of ventricular stiffness, was elevated in DSHR animals [31.5 ± 1.9 *vs*. 19.4 ± 0.9 (UNX), *p* < 0.0001], and treatment with CST-WT rescued this phenotype (20.4 ± 1.8, *p* < 0.0001 *vs*. DSHR), while treatment with CST-Ser did not exhibit a significant reduction (27.5 ± 0.6, *p* = 0.33 *vs*. DSHR). Patients with diastolic heart failure display higher passive stiffness [33]. Moreover, animals treated with CST-Ser displayed higher stiffness as compared to the normotensive animals (*p* = 0.002) (Figure 5E), thereby demonstrating the inability of CST-Ser to restore myocardial function in DSHR.

### Effect of CST-WT and CST-Ser peptides on cardiac inflammation

**Figure 6:**
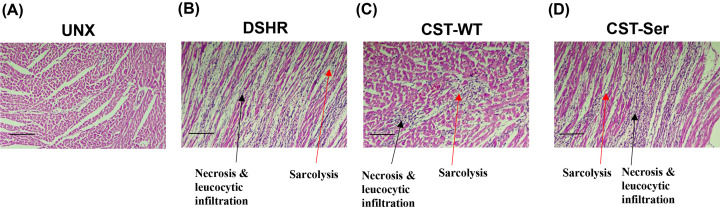
Effect of CST-WT and CST-Ser peptides on cardiac inflammation Cardiac tissue inflammation markers, such as lymphocytic and leucocytic infiltration along with sarcolysis, were histologically analyzed, after staining with hematoxylin and eosin. Tissue sections were analyzed from at least 5 animals per experimental group and representative images are shown. Scale bar: 250 micrometer. Sections were graded as *NAD* (no abnormalities were detected), *minimal* (1/+), *mild* (2/++), *moderate* (3/+++), and *marked* (4/++++), depending upon the extent of inflammation. (**A**) UNX hearts (NAD). (**B**) The black arrow indicates areas with the presence of leukocytic infiltration and necrosis, while the red arrow depicts sarcolysis in the DOCA-salt-treated heart tissue (Grade 4). Tissues from (**C**) CST-WT-treated animals displayed regions of focal necrosis and lymphocytic infiltration (black arrow; Grade 1) and (**D**) CST-Ser-treated animals exhibited enhanced lymphocytic infiltration (black arrow) and sarcolysis (red arrow) (Grade 3). UNX: control animals subjected to unilateral nephrectomy and administered with vehicle; DSHR: animals subjected to unilateral nephrectomy and DOCA-salt treatments; CST-WT: DSHR animals treated with CST-WT peptide; CST-Ser: DSHR animals treated with CST-Ser peptide.

H&E staining of cardiac tissue sections showed profound inflammation (Grade 4) in the DSHR group, as evident from the presence of extensive myocardial necrosis, with resultant loss of sarcoplasm. These tissues also exhibited moderate scarring and lymphocytic infiltration in the necrotic area, whereas no such abnormalities were detected in cardiac tissues of the UNX rats (Figure 6A,B).

Treatment with CST-WT considerably reduced the infiltration of lymphocytes in the necrotic area, with observation of minimal multifocal myocytic necrosis (Grade 1) (Figure 6C). On the other hand, cardiac tissues of CST-Ser-treated animals displayed multifocal areas of myocytic necrosis, with resultant loss of sarcoplasm (Figure 6D). Moreover, moderate scarring and lymphocytic infiltration were also observed in the necrotic area (Grade 3), suggesting reduced anti-inflammatory potential of CST-Ser.

## Discussion

### The Gly364Ser variation in the CST peptide: BP dysregulation and other pathophysiological implications

The Gly364Ser variation is the most common one among the human variants of the CST peptide [11]. Analysis of multiple human populations revealed that the Ser allele, *in general*, confers higher BP (Figure 1). In line with this observation, different GWASes also revealed positive association of the Ser allele with several risk factors for CVDs, chronic kidney disease, and diabetic kidney disease, in addition to coronary artery disease (Figure S4). CST-WT administration resulted in marked reduction of the SBP, DBP, and MAP in the DSHR animals but CST-Ser was far less effective. Treatment with CST-WT caused reduction in PP and dicrotic notch pressure to almost control levels, as evidenced by the lack of significance between the control and CST-WT groups. Although SBP, DBP, and MAP values upon treatment of CST peptides were significantly different with respect to the control group, CST-WT was nevertheless far more effective than CST-Ser in reducing BP parameters (Figure 2), in line with the observations in the human populations [10,11]. However, this study does not intend to claim that CST-WT can prevent or reverse hypertension altogether, although these endogenous peptides act as a BP-lowering agents. Of note, effect of the CST-WT peptide was observed during both systole and diastole phases, indicating improved ventricular polarization and depolarization phases, respectively. These findings also indicate improved aortic conduction and ventricular contraction implying better cardiac function in the animals treated with CST-WT peptide.

### Impact of the CST-WT and CST-Ser peptides on cardiac function

CST treatment was reported to enhance heart rate under basal conditions in the Langendorff-perfused rat heart [41]. In the present study, CST-WT was able to lower heart rates in hypertensive animals significantly close to the control level, but CST-Ser was not able to exert significant reduction in heart rate (Figure 3A). This is reminiscent of the mitigation of heart rate upon CST administration in *Chga*-KO mice subjected to immobilization stress. Prolonged P-wave durations are observed in hypertensive subjects [34]. CST-WT was found to be more effective in blunting the P-duration than CST-Ser (Figure 3B), indicating that CST-WT treatment causes better atrial conductions and atrial myocytes contraction pattern, suggesting an improvement in atrial functions. In models with variable expression of CHGA (sufficient, Hum*CHGA*31 *vs*. insufficient, Hum*CHGA*19, exhibiting lower CST levels), the duration of QRS and QTc intervals were significantly higher in the Hum*CHGA*19 animals than in the Hum*CHGA*31 animals [35], thus asserting the necessity of optimal CHGA/CST levels in governing cardiac electrical conduction. Indeed, both CST-WT and CST-Ser were successful in lowering the QRS interval (Figure 3C). Interestingly, CST-WT treatment brought back the QTc interval (a heart rate-independent ventricular repolarization marker for future cardiac failure prediction) to almost near the control level, indicating a significant reduction in the future chances of cardiac failure, but CST-Ser was not as effective in reducing the QTc interval (Figure 3D). Of note, a GWAS in the European population demonstrated a positive effect of the G364S variant with QTc interval [36] (accessed from the Common Metabolic Disease Knowledge Portal on Sept 03, 2024).

Consistent with observations in humans [37], the RR interval was reduced in the DSHR group. CST-WT was able to rescue the RR interval (Figure 3E), reminiscent of diminished RR intervals in Hum*CHGA*19 animals, as compared to Hum*CHGA*31 animals [35], while CST-Ser could not. There was a drastic decline in the ratio of sympathetic (LF) and parasympathetic (HF) activities, which serves as a measure of symapathovagal balance, in the hypertensive rats treated with CST-WT, while CST-Ser treatment was not as potent (Figure 3F). Heightened sympathetic activity is a key driver of hypertension [38], and a significant decrease in vagal activity is believed to contribute to the perturbation of the sympathovagal balance in the hypertensive milieu [39]. A higher LF/HF ratio suggests enhanced sympathetic tones [40]. Therefore, a reduction in the LF/HF ratio after CST administration reflects the sympatho-inhibitory activity of CST. Our results provide *in vivo* evidence of inefficient inhibition of sympathetic tone by CST-Ser which is consistent with the diminished BP-lowering effect of this peptide.

A recent report demonstrated the cardioprotective roles of CST in a transverse aortic constriction/DOCA mouse model of diastolic dysfunction and heart failure with preserved ejection fraction [8]. The effect of CST on cardiac performance was previously reported: CST-WT reduced the LV pressure, +(dP/dT)_max_, and –(dP/dT)_max_ under basal and isoproterenol-induced conditions in Langendorff-perfused hearts [41]. While CST-Ser did not modulate contractility under basal conditions, it could significantly modulate cardiac parameters under isoproterenol-stimulated conditions [41]. CST-WT was more potent in counteracting isoproterenol-stimulated positive inotropism than CST-Ser, while surprisingly, the reverse was true in the case of isoproterenol-induced lusitropism and endothelin-1-induced coronary constriction [41]. We observed that CST-WT blunted the DOCA-salt-induced increase in the Max and Mean LV pressures to almost control levels whereas CST-Ser was far less effective (Figure 4A,B). The widely-used indices of LV contractility [*viz*., (dP/dT)_max_ and contractility index] were diminished in the DSHR group, suggesting impaired contractility. Administration of CST-WT resulted in an appreciable enhancement of these parameters; on the other hand, CST-Ser was much less effective in the case of (dP/dT)_max_ and not effective in the case of contractility index (Figure 4C,D). To our knowledge, the current study is the first report to investigate the effect of CST on ventricular wall stress.

The significant reduction of the aggravated Min LV pressure of the hypertensive animals upon CST-WT administration, but not CST-Ser administration (Figure 5A), is in agreement with a previous report where elevated Min LV pressure was observed in DSHR animals with LV dysfunction with preserved ejection fraction subjected to elevated arterial pressure [42]. (dP/dT)_min_, a measure of the change in pressure during relaxation, which decreased in hypertension, was boosted upon administration of CST-WT (by ∼4.3-fold with respect to DSHR); on the other hand, CST-Ser treatment could increase it by ∼2.1-fold as compared to DSHR animals (Figure 5B), showing profound reduction in the efficacy of the CST-Ser peptide. Similarly, alleviation of end-diastolic pressure and the relaxation time constant Tau was observed upon administration of CST peptides, although CST-WT was much more effective (Figure 5C,D). In addition, CST-WT treatment mitigated the passive diastolic stiffness in the hypertensive animals to the control level whereas CST-Ser did show any significant reduction (Figure 5E). Thus, our findings suggest markedly reduced cardioprotective effects of the CST-Ser peptide in DOCA-salt hypertensive rats as compared to the CST-WT peptide.

### Anti-inflammatory effects of CST-WT and CST-Ser peptides

The immune system plays striking roles in several cardiovascular pathologies, including hypertension [43]. The anti-inflammatory nature of CST has been demonstrated [44], and further validated by observations of an exacerbated inflammatory profile and fibrosis in the hearts of CST-KO mice. Furthermore, CST supplementation reduced monocyte recruitment and macrophage infiltration in CST-KO hearts [18]. In agreement with these observations, we observed a marked reduction in sarcolysis and lymphocytic infiltration in the hearts of hypertensive animals treated with CST-WT, as compared to the abundant areas of myocytic necrosis, along with moderate scarring and leukocyte infiltration seen in DOCA-salt-treated animals (Figure 6B,C). The effects of CST-Ser, however, were not as pronounced (Figure 6D), suggesting impaired potential to mediate anti-inflammatory effects. Consistent with this finding, plasma biochemistry analysis showed marked reduction of DOCA--induced C-reactive protein (CRP) level by CST-WT whereas CST-Ser showed minimal effect on plasma CRP level (data not shown). This observation holds translational potential, as knowledge of the impaired inflammatory response in carriers of this variant can be exploited to devise novel therapeutic strategies. The minimal inflammatory damage caused by CST-WT could be explained by the reduced hemodynamic load, and consequently reduced pressure on the heart and blood vessels. Moreover, heightened sympathetic tone has been shown to contribute to inflammatory responses [45]. The enhanced ability of CST-WT to suppress sympathetic activation could result in mitigated inflammatory responses, as compared to those seen in case of CST-Ser.

### Differential actions of CST-Ser peptide on hypertension: mechanistic insights

What is the mechanistic basis for the diminished efficacy of the CST-Ser peptide (as compared to the CST-WT peptide) in hypertension? Figure S5 represents the different signalling routes through which CST peptides modulate cardiac pathophysiology. CST-Ser is less potent than CST-WT as an antagonist of nAChR, as evidenced by its impaired ability to blunt nicotine/nAChR-mediated processes, *viz*., catecholamine release, desensitization of catecholamine release, gene transcription, inward currents, and intracellular calcium levels [10,46,47]. Computational analysis revealed that CST-Ser had weaker binding affinity and ability to occlude the extracellular vestibular region of nAChR [46], thus dictating altered interactions. Endothelial dysfunction plays a salient role in the hypertensive pathophysiology. Our group demonstrated that CST-Ser diminishes endothelial nitric oxide synthase activity, and causes a concomitant decrease in endothelial NO production, as compared to CST-WT, thereby conferring a higher risk of hypertension to the carriers of this genotype [11]. NO plays key roles in governing spontaneous tone in DOCA-salt rats [48] and diminished endothelial nitric oxide synthase expression has been observed in the mesenteric arteries of DOCA-salt rats [48,49]. Thus, the NO pathway could be a major target of CST action in the DOCA-salt model.

Studies show that the cardio-modulatory effects of CST are mediated through α/β- adrenoreceptors, Gi/o proteins, eNOS/NO/cGMP/PKG axis, PLN/SERCA2a, PI3K/Akt, ERK, GSK-3β, or type-2 muscarinic acetylcholine receptor, under basal and pathophysiological conditions [50–52]. Further studies need to be carried out to unearth the involvement of these signalling molecules in CST-mediated cardioprotection in the DOCA-salt model.

Expression quantitative trait loci data from Phenoscanner [53,54] revealed that the Gly364Ser variant modulated the expression of the neighboring genes of CHGA, *viz*., UNC79 (β = –1.034, *p* = 2.3e^–05^) and GOLGA5 (*p* = 8.3e^–04^). Intriguingly, Chga, Unc79, and Golga5 are harbored in three BP quantitative trail loci found on chromosome 6 in rats (Figure S6), suggesting that this variant could also impact BP by governing the expression of UNC79 and GOLGA5. The role of these genes in BP modulation remains to be established and warrants further investigation.

## Conclusions

CST is a CHGA-derived peptide that has gained prominence as an endogenous anti-hypertensive and cardioprotective peptide. While CST-WT showed profound anti-hypertensive effects in the DOCA-salt model of hypertension CST-Ser was far less effective in lowering the BP parameters. CST-WT was also more effective in improving the cardiac parameters in the DOCA-salt model as compared to CST-Ser. To our best knowledge, this is the first study to comprehensively investigate the impaired cardioprotection conferred by the naturally-occurring variant of CST peptide (*viz.*, CST-Ser) in an animal model of hypertension. The diminished efficacy of the CST-Ser peptide (as compared to the CST-WT peptide) for reducing high BP and multiple cardiac parameters in this hypertensive animal model may be attributed to the alterations in the peptide structure that ultimately alter interactions with its cognate receptors. These insights have implications in a clinical setting, to facilitate development of prognostic tools and tailored therapeutic strategies.

## Supplementary Material

Supplementary Figures S1-S6

## Data Availability

All data from this study are available from the corresponding authors upon reasonable request.
