# Supplementary material for: The physiological anti-hypertensive peptide catestatin and its common human variant Gly364Ser: differential cardiovascular effects in a rat model of hypertension
Source: Biosci Rep. 2024 Dec 12;44(12):BSR20241433. doi: 10.1042/BSR20241433 (PMC11667095; doi:10.1042/BSR20241433)
Supplement: Supplementary Figures S1-S6 [file BSR-2024-1433_supp.pdf]

Supplementary Material

The physiological anti-hypertensive peptide catestatin and its common human variant Gly364Ser: differential cardiovascular effects in a rat model of hypertension

by

Jitesh Singh Rathee[1*], Dhanya R. Iyer[2], Malapaka Kiranmayi[2], Samarasimha Reddy[3], V.V. Sureshbabu[3], and Nitish R. Mahapatra[2*]

from the

[1] Bio-Organic Division, Bhabha Atomic Research Centre, Mumbai 400085, India

[2] Department of Biotechnology, Bhupat and Jyoti Mehta School of Biosciences, Indian Institute of Technology Madras, Chennai 600036, India

[3] Department of Studies in Chemistry, Central College Campus, Bangalore University, Dr. B.R. Ambedkar Veedhi, Bangalore 560001, India

*Correspondence to:

Dr. Nitish R. Mahapatra, FNA, FNASc, FAHA
Department of Biotechnology
Bhupat and Jyoti Mehta School of Biosciences
Indian Institute of Technology Madras
Chennai 600036, India
E-mail: nmahapatra@iitm.ac.in
Tel: 91-44-2257-4128

or

Dr. Jitesh Singh Rathee
Bio-Organic Division
Bhabha Atomic Research Centre
Mumbai 400085, India
E-mail: ratheej@barc.gov.in
Tel: 91-22-2559-0268

SUPPLEMENTARY FIGURES

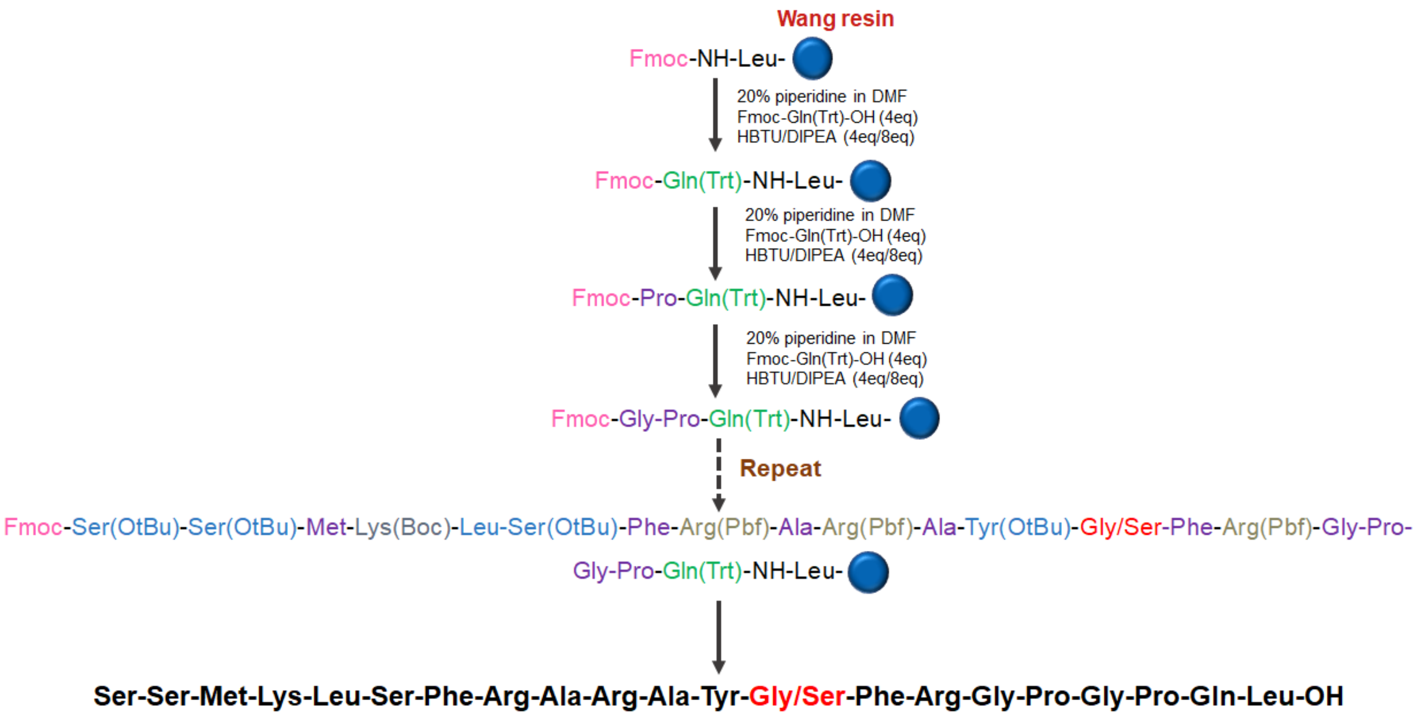

Figure S1. Schematic of the solid-phase synthesis process carried out for CST-WT and CST-Ser peptides. The steps carried out for the synthesis of CST-WT/CST-Ser peptides are shown. The position of the Glycine or Serine residue in the peptide sequence are shown in red font. HBTU: 2-(1H-benzotriazol-1-yl)-1,1,3,3-tetramethyluronium hexafluorophosphate; DIPEA: N,N-diisopropylethylamine.

A.

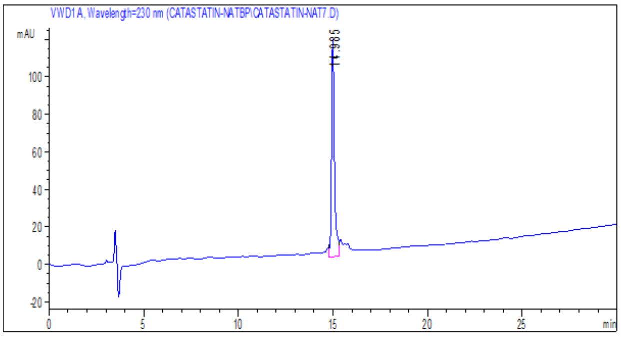

B.

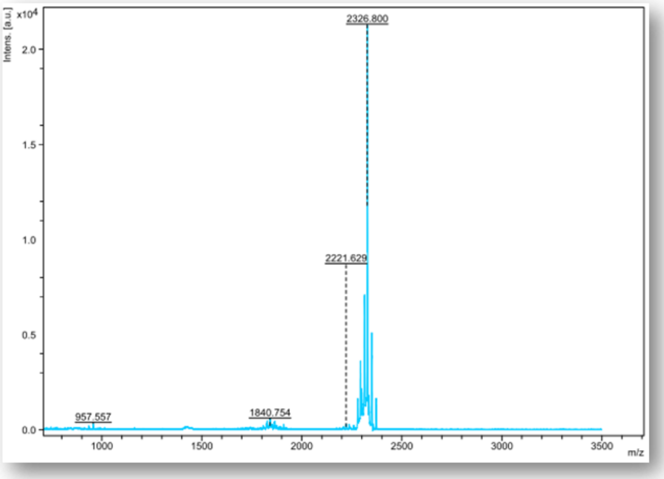

Figure S2. Chromatography and mass spectroscopy profiles of CST-WT peptide. Following solid phase synthesis, the quality and authenticity of CST-WT peptide were assessed using high-performance liquid chromatography (panel A) and mass spectroscopy (panel B).

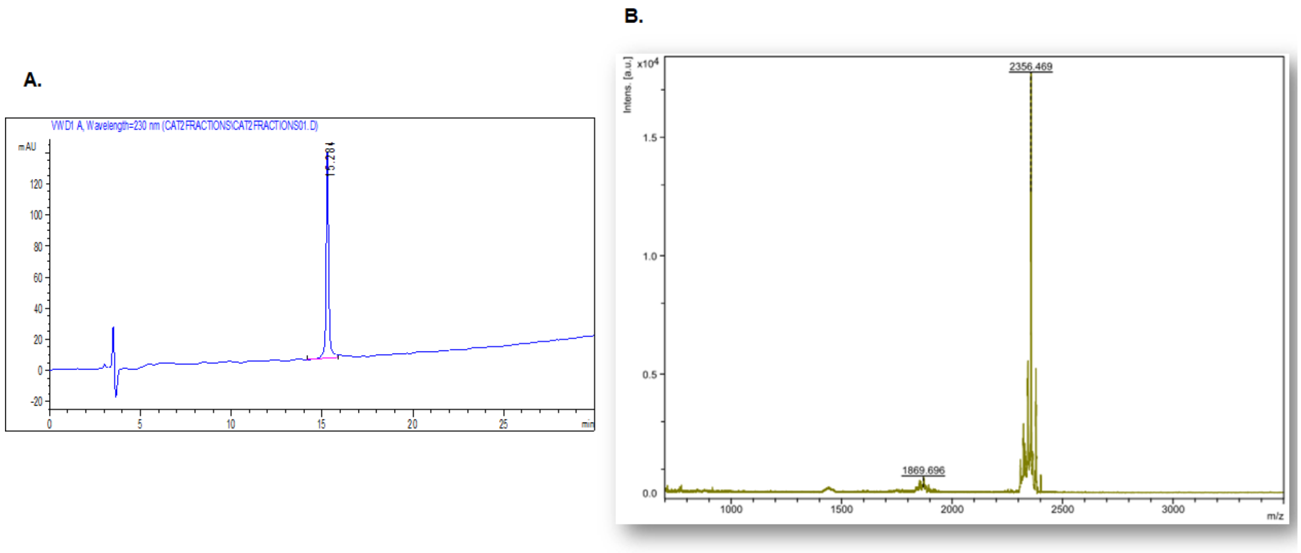

Figure S3. Chromatography and mass spectroscopy profiles of CST-Ser peptide. Following solid phase synthesis, the quality and authenticity of CST-Ser peptide were assessed using high-performance liquid chromatography (panel A) and mass spectroscopy (panel B).

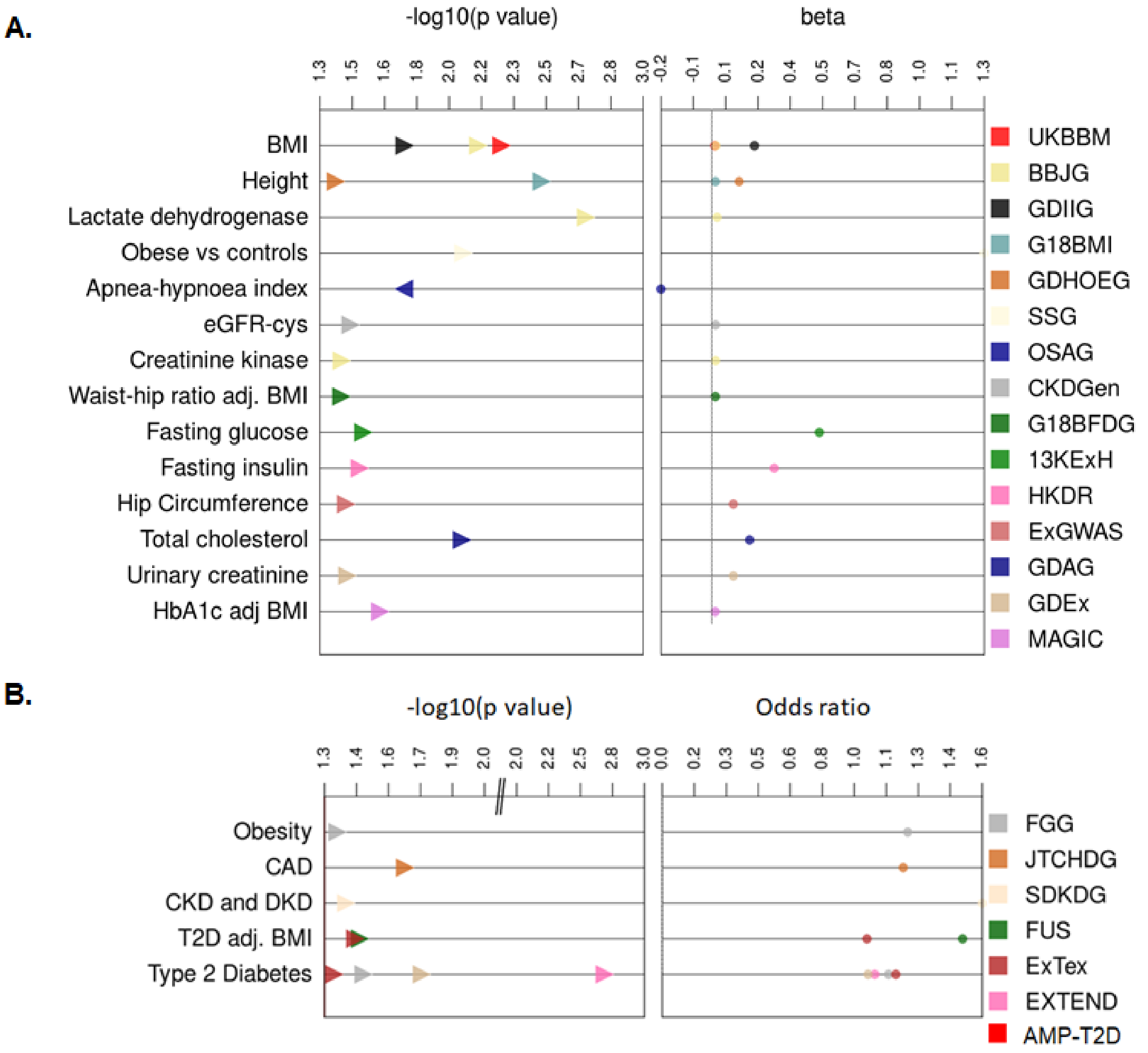

Figure S4. Cardiometabolic associations of the CST-G364S (rs9658667) variant found in genome-wide association studies (GWASes). Associations of the CST-G364S (rs9658667) variant with cardiometabolic (A) traits and (B) disorders found in various GWASes were visualized using the PheWAS-View tool (Pendergrass et al 2012). Association data were obtained from the Common Metabolic Diseases Knowledge Portal (cmdkp.org) (rs9658667 Variant page 2021 Dec 1; https://hugeamp.org/variant.html?variant=rs9658667). The studies from which these associations were obtained are: UKBBM-GIANT-UK Biobank GWAS meta-analysis (n=484680); BBJG-BioBank Japan GWAS (n=158284 for BMI, n=126319 for lactate dehydrogenase, and n=106080 for creatinine kinase); GDIIG-GoDarts Illumina Infinium GWAS (n=1884); G18BMI-GIANT 2018 BMI Height exome chip analysis (n=718734); GDHOEG-GoDarts Illumina Human OMNIExpress GWAS (n=2902); SSG-SCOOP vs STILTS GWAS (n=4752); OSAG-Obstructive sleep apnea GWAS:Europeans (n=1348); CKDGen-CKDGen 1000G GWAS-eGFR associations (n=21070); G18BFDG-GIANT 2018 Body Fat Distribution exome chip analysis: Europeans (n=250294); 13KExH-13K exome

sequence analysis: Hispanics (n=853); HKDR-Hong Kong Diabetes Register GWAS (n=321); ExGWAS-EXTEND GWAS (n=7159 for hip circumference and n=4492 for type-2 diabetes); GDAG-GoDarts Affymetrix GWAS (n=2917); GDEx-GoDarts exome chip analysis (n=4863); FGG-FinnGen GWAS (n=11743 for obesity and n=38850 for type-2 diabetes); JTHCDG-Joint T2D-CHD GWAS (n=236574); SDKDG-SUMMIT Diabetic Kidney Disease GWAS: subjects with T2D (n=1448); MAGIC-MAGIC 2021 glycemic traits GWAS: East Asians (n=31263); FUS-FUSION exome chip analysis (n=3339); ExTex-ExTexT2D exome chip analysis (n=175703 for type-2 diabetes adj. BMI and n=217209 for type-2 diabetes); AMP-T2D AMP T2D-GENES exome sequence analysis: East Asians (n=12177). BMI, body mass index.

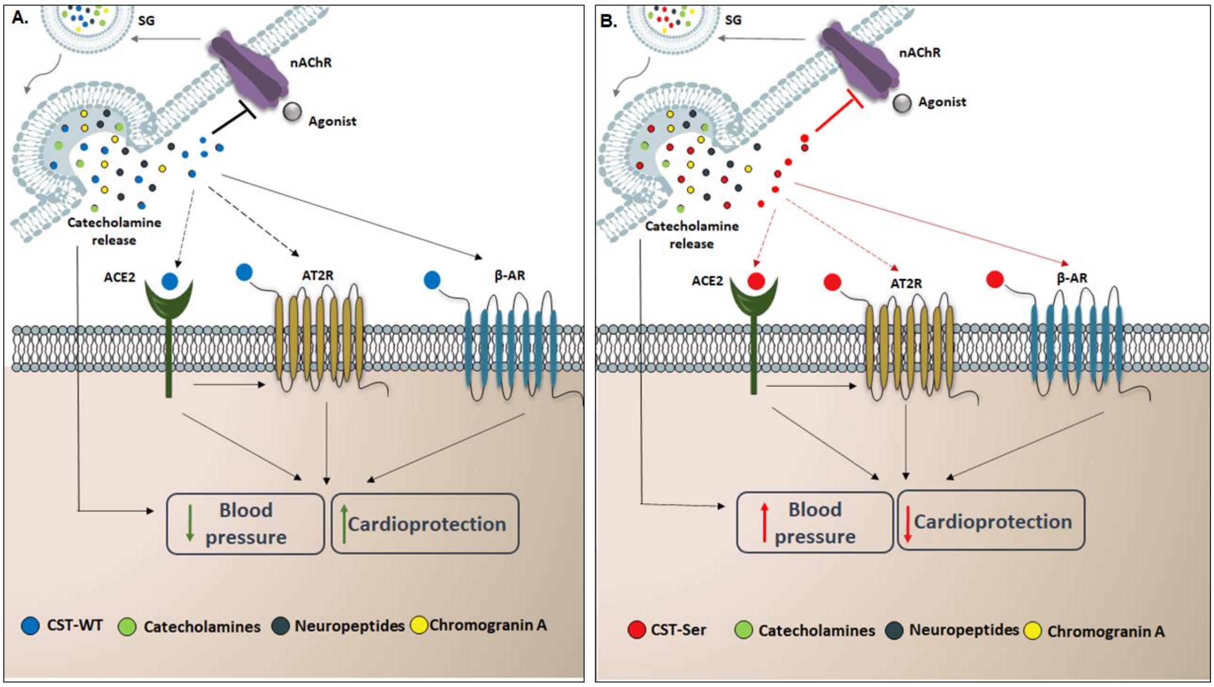

Figure S5. Schematic illustration of potential mechanisms of action of CST peptides. (A) The left panel depicts the receptors through which CST-WT possibly exerts its anti-hypertensive and cardioprotective effects. The nicotine cholinergic-inhibitory role of CST is believed to underlie its catecholamine release-inhibitory action. CST-WT is known to interact with the β_2-adrenergic receptor and mediate enhanced production of endothelial nitric oxide. CST-WT peptide may also exert its anti-hypertensive and cardioprotective effect via renin-angiotensin system (RAS) pathway since the impact of CST-WT peptide on RAS signaling has been previously investigated in the context of coronary artery disease (Chen et al. 2019). (B) The right panel depicts the altered actions of CST-Ser on its cognate receptors (indicated by red arrows). Altered interactions with nicotinic acetylcholine receptor (nAChR) and β-adrenergic receptor (β-AR) may account for the reduced potency of CST-Ser in lowering blood pressure and offering cardioprotection. ACE2, angiotensin converting enzyme 2; AT2R, angiotensin receptor type 2; SG, secretory granule.

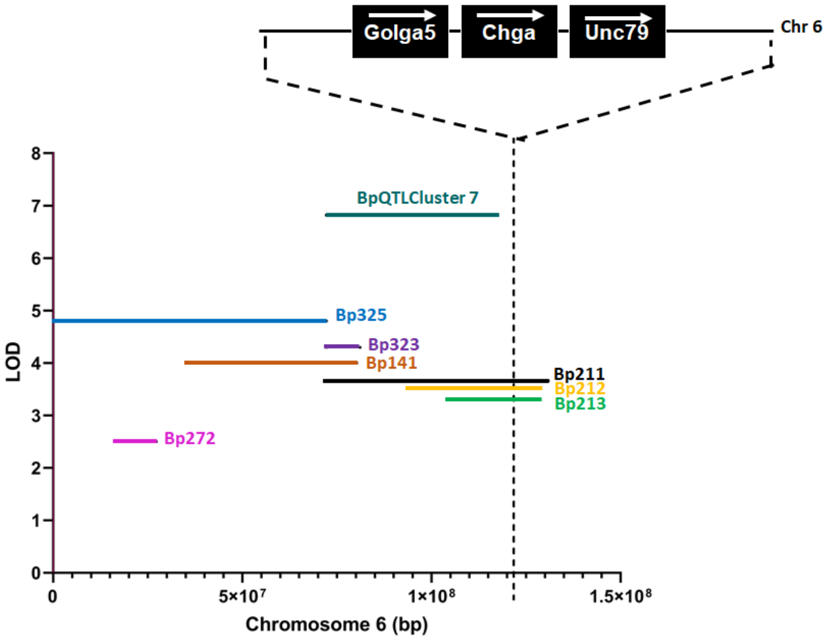

Figure S6. Localization of chromogranin A gene (*Chga*) in the blood pressure quantitative trait loci on rat chromosome 6. This figure depicts the blood pressure quantitative trait loci on chromosome 6 in the rat. The X-axis indicates the length of the chromosome, while the Y-axis denotes the logarithm of the odds (LOD) score. *Chga* and its neighboring genes whose expressions are altered as a result of the CST-Ser variant (*viz.* Unc79 and Golga5), are harbored in three blood pressure quantitative trait loci: Bp211, Bp212, and Bp213 (Source: Rat Genome Database, https://rgd.mcw.edu/)

REFERENCES

1. Pendergrass SA, Dudek SM, Crawford DC, Ritchie MD. Visually integrating and exploring high throughput Phenome-Wide Association Study (PheWAS) results using PheWAS-View. BioData Min. (2012) 5:5. doi: 10.1186/1756-0381-5-5.
2. Chen Y, Wang X, Yang C, Su X, Yang W, Dai Y, Han H, Jiang J, Lu L, Wang H, Chen Q, Jin W. Decreased circulating catestatin levels are associated with coronary artery disease: The emerging anti-inflammatory role. Atherosclerosis. (2019) 281:78-88. doi: 10.1016/j.atherosclerosis.2018.12.025.
